# Causal relationships between dietary factors and spinal diseases: a univariable and multivariable Mendelian randomization study

**DOI:** 10.3389/fnut.2025.1437484

**Published:** 2025-03-14

**Authors:** Yi-Qi Chen, Zhen-Ya Chen, Zheng-Qi Song, Hai-Bo Liang, Yi-Jun Li, Hong Su, Hai-Ming Jin, Xue-Qin Bai

**Affiliations:** ^1^Department of Radiology, The First Affiliated Hospital of Wenzhou Medical University, Wenzhou, China; ^2^The Second Clinical Medical College, Wenzhou Medical University, Wenzhou, Zhejiang, China; ^3^The First Clinical Medical College, Wenzhou Medical University, Wenzhou, Zhejiang, China; ^4^Department of Orthopaedics, The Second Affiliated Hospital and Yuying Children’s Hospital of Wenzhou Medical University, Wenzhou, China

**Keywords:** spinal diseases, degenerative spinal diseases, food liking, food intake, Mendelian randomization

## Abstract

**Background:**

Spinal diseases and their associated symptoms are prevalent across all age groups, and their incidence severely affects countless individuals’ quality of life. The role of daily habits in the progression of these diseases is increasingly emphasized in research. Moreover, there are reports suggesting associations between dietary factors and the onset of spinal diseases. However, the exact causal relationship between dietary factors and spinal diseases has not been fully elucidated.

**Methods:**

We obtained GWAS data on 16 dietary intake and 187 dietary likings from the UK Biobank, and GWAS data on 23 types of spinal disorders from FinnGen R10. The analysis of causal effects was conducted using the Inverse Variance Weighted (IVW) test, and to ensure robustness, MR-Egger, Weighted median, and Bayesian weighted Mendelian randomization (BWMR) were utilized to validate the direction. Sensitivity analysis was conducted using the Cochran Q test and MR-Egger intercept test. Additionally, Multivariable MR (MVMR) was employed to examine the independent effect of alcohol intake frequency.

**Results:**

In summary, our study identified statistically significant causal associations between four dietary intake and 10 dietary linkings with various spinal disorders through univariable MR, with degenerative spinal changes showing the most significant dietary influence. Alcohol intake was identified as the primary risk factor, with other risk factors including poultry intake and likings for various types of meat. Protective factors mainly included intake and liking of fruits and vegetables. Additionally, various supplementary analytical methods along with heterogeneity and pleiotropy tests have confirmed the robustness of our results. To avoid the interference of diet-related diseases, multivariable MR analysis was conducted, showing that the incidence of cervical disc disorders may be influenced by gout, diabetes, and hypertension.

**Conclusion:**

This study indicates a potential causal relationship between dietary factors and the risk of spinal disorders, providing insights for the early detection and prevention. However, the specific pathogenic mechanisms require detailed basic and clinical research in the future.

## 1 Introduction

Spinal disease (SPD) is one of the most common diseases across all age groups, imposing significant and measurable burdens on affected patients and healthcare economies (1). SPD encompasses a variety of conditions, including degenerative spinal diseases, spinal deformities, and inflammatory, infectious, and immune-related spinal diseases, as well as disorders of the spinal cord and associated neural symptoms. The spine, a complex structure, provides critical protection and support for the spinal cord across various body positions and postures (2). As individuals age, the spine endures excessive loads, leading to widespread degenerative abnormalities affecting both the bony structures and intervertebral discs (2, 3). Degeneration of intervertebral discs, known as intervertebral disc degeneration (IVDD), serves as the pathological basis for many degenerative spinal conditions. This degeneration is characterized by a gradual reduction in proteoglycan and water content within the nucleus pulposus of the discs (4, 5). Symptoms arising from IVDD include low back pain, disc herniation, spinal stenosis, and cervical spondylosis (6). With the progression of IVDD, the discs may rupture, potentially compressing nerves and causing lumbar radiculopathy and sciatica (7, 8). Although the exact mechanisms leading to IVDD have not been fully established, several contributing factors are recognized, including genetic predisposition, age, obesity, smoking, trauma, and abnormal non-physiological mechanical loads (9–11). Current clinical treatments for IVDD primarily consist of conservative and surgical options that alleviate symptoms and reduce pain but do not reverse the condition, highlighting the need for early intervention to slow disease progression (6). Regarding spinal deformities, these are abnormalities in the alignment, formation, or curvature of one or more parts of the spine (12). Common spinal deformities include scoliosis, kyphosis, lordosis, spinal instability, spinal osteochondrosis, and kissing spine. In adolescents, persistent idiopathic scoliosis is prevalent, whereas in the elderly, degenerative scoliosis and kyphosis are more common (13, 14). Spinal osteochondrosis, also known as Scheuermann’s disease, is the second most common growth-related spinal deformity. It affects approximately 4–6% of the general population and 1–8% of adolescents, with patients presenting with back pain at twice the prevalence seen in the general population, and its prevalence may be increasing (15, 16). Inflammatory, infectious, and immune-related spinal diseases, such as discitis, ankylosing spondylitis, and spinal enthesopathy, can lead to inflammatory back pain and excessive spinal bone formation (17). Developmental and degenerative anomalies affecting spinal substructures can compress the spinal cord and associated neural elements, leading to conditions such as myelopathy and cauda equina syndrome, which in turn cause neurological complications including pain and paralysis, significantly diminishing the patient’s quality of life and life expectancy (18).

Dietary habits refer to the long-term patterns and behaviors related to food consumption that individuals develop and maintain in their daily lives. Positive dietary behaviors are crucial strategies for maintaining personal health. In contrast, poor dietary choices characterized by excessive consumption of processed foods, added salts, and unhealthy fats are associated with an increased risk of chronic diseases such as obesity, diabetes, and cardiovascular diseases (19, 20). As society has evolved, the drivers of dietary behaviors have shifted from the necessity of consuming merely available food to choosing from a diverse selection of options (21). Liking for the certain food reflect an individual’s hedonic response to food, which is strongly correlated with the amount of food consumed, and this liking is genetically more stable than the actual quantity of food intaked (21, 22). Dietary behaviors are importantly linked to spinal health; a systematic review has shown that plant-based diets may alleviate chronic musculoskeletal pain, whereas higher intake of protein, fats, sugars, and calories are positively correlated with pain intensity (23). Furthermore, studies indicate that a daily intake of fruits and vegetables is associated with a lower risk of lower back pain (24). Caffeine intake might be a risk factor for IVDD, especially if the intervertebral disc is already damaged or degenerated (25). Several studies suggest that the development of degenerative spinal diseases may result from the interplay between genetic and environmental factors. Specific genetic variations may influence the structure and function of bones and cartilage, and these effects can be further modulated by environmental factors such as micronutrient intake. One example is the polymorphism of the VDR gene, where the ApaI A allele has been associated with the occurrence of IVDD, potentially affecting disc metabolism and degeneration by regulating the vitamin D signaling pathway. This highlights the critical role of gene–environment interactions in the progression of spinal diseases (26, 27). While dietary patterns play a significant role in musculoskeletal health, it is important to note that the conclusions of these studies are mostly correlational and do not establish causality.

Mendelian randomization (MR) analysis is an analytical method that utilizes genetic instrumental variables from genome-wide association studies (GWAS) to assess the causal relationship of specific exposures on outcomes (28), mirroring the principles of randomized controlled trials (RCTs) (29). Because single nucleotide polymorphisms (SNPs) are randomly allocated at conception, MR analysis can effectively eliminate confounding factors and reverse causation (29). Previous studies have revealed causal relationships between dietary intake and low back pain using Mendelian randomization analysis; however, these studies did not explore dietary likings or other spinal diseases. To address this gap, this study employs MR analysis to investigate the causal relationships between dietary factors (16 dietary intake and 187 dietary likings) and various spinal diseases, providing a basis for designing more effective and precisely targeted dietary intervention plans to prevent the progression of specific spinal diseases.

## 2 Methods

### 2.1 Study design

**Figure 1:**
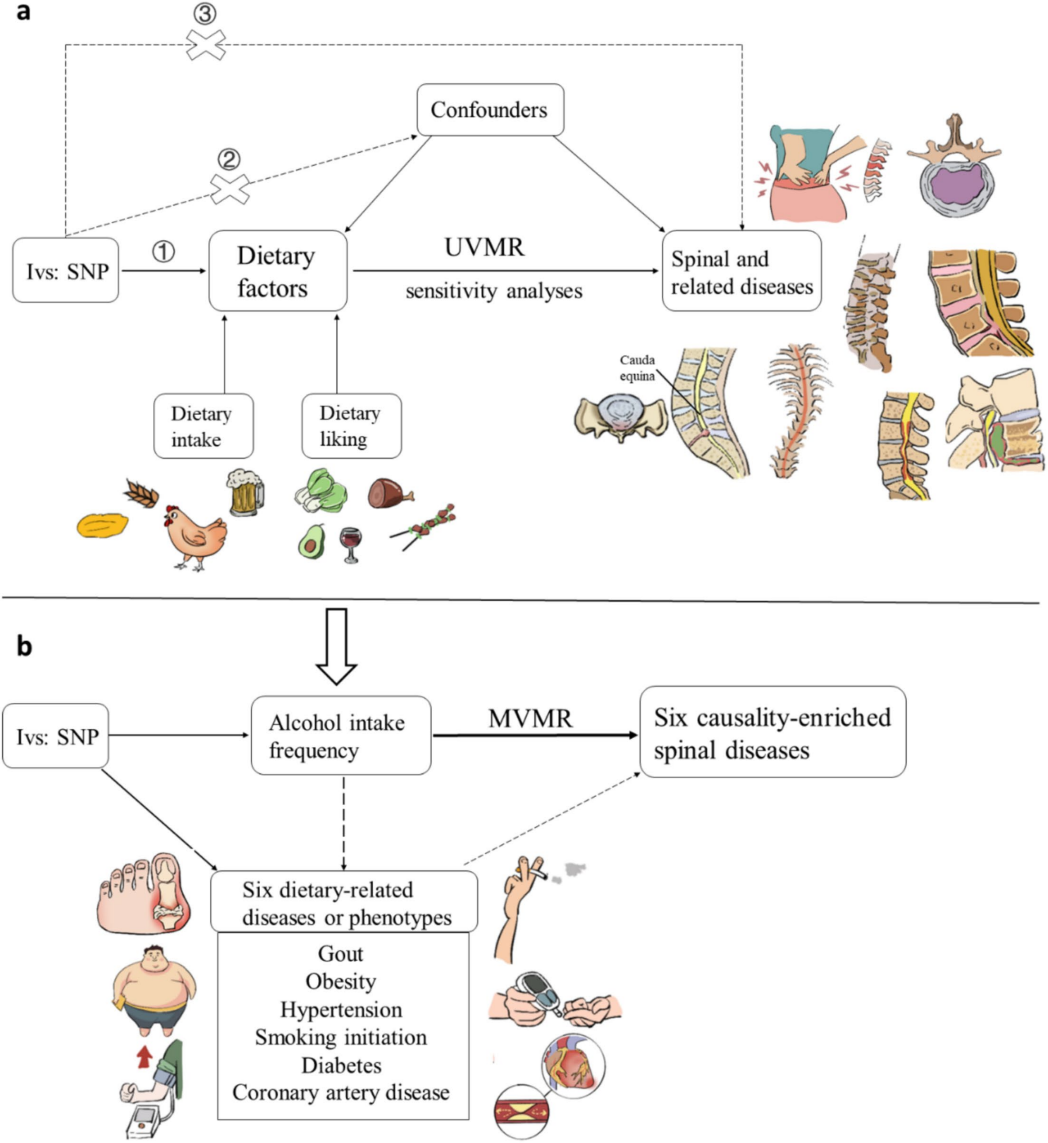
Schematic representation of the **(a)** univariable Mendelian randomization analysis and **(b)** multivariable Mendelian randomization analysis. Three key assumptions of MR: (1) genetic variants must be associated with exposures; (2) genetic variants must not be associated with confounders; (3) genetic variants must affect outcomes only through exposures, not through other pathways.

We first utilized a two-sample Mendelian randomization (MR) analysis to examine the causal effects of dietary intake and likings on the risk of spinal and related diseases. In this analysis, single nucleotide polymorphisms (SNPs) strongly associated with 16 dietary intake and 187 dietary likings were used as instrumental variables (IVs). The MR analysis was based on three core assumptions: (1) the Correlation assumption, stipulating that SNPs are closely related to the exposure (dietary factors); (2) the Independence assumption, asserting that SNPs are not associated with any potential confounders, such as obesity or tobacco use; (3) the Exclusion assumption, positing that SNPs influence the outcome (spinal and related diseases) solely through the exposure (dietary factors) (Figure 1a). Furthermore, to identify potential confounders and mediators, we included diseases and phenotypes significantly related to diet based on previous research, and selected important causal relationships in the two-sample MR analysis for multivariable Mendelian Randomization (MVMR) analysis (Figure 1b).

### 2.2 GWAS data source

In exploring the impact of dietary factors, we utilized two sets of genome-wide association studies (GWAS) data, specifically focusing on 16 dietary intake habits and 187 dietary likings (21). The 16 dietary intake GWAS data we included encompassed alcohol intake frequency, beef intake, bread intake, cereal intake, cheese intake, coffee intake, cooked vegetable intake, dried fruit intake, fresh fruit intake, lamb/mutton intake, oily fish intake, non-oily fish intake, pork intake, poultry intake, salad/raw vegetable intake and tea intake. These GWAS summary-level data were obtained from the UK Biobank (UKB) and are accessible for download via the Integrative Epidemiology Unit (IEU) open GWAS project.^1^ The 16 ordinary dietary intake information was collected through a touchscreen questionnaire, with detailed specifics provided in Supplementary Table S1. Further details are available for public inquiry on the UKB website.^2^ The GWAS summary data of food liking was obtained from 161,625 participants from the UK Biobank. The degree of liking for each specific food was assessed using a 9-point scale and was categorized into three dimensions: “Highly-palatable,” “Acquired,” and “Low-caloric” (21). The information on food liking is also collected by questionnaires, with participants rating their liking for each food item on a scale of nine levels, ranging from “Extremely dislike” to “Extremely like.” Details of the questionnaire can be found at https://biobank.ndph.ox.ac.uk/showcase/showcase/docs/foodpref.pdf. Details regarding the intake and liking of each food item can be found in the original article and research. The basic information of GWAS data for a total of 203 dietary factors used as exposures for mendelian randomization in this study is presented in Supplementary Tables S2.

The GWAS summary results for spinal and related diseases were acquired from the FinnGen R10 database.^3^ We endeavored to collect the spine and its associated conditions data from the FinnGen R10 GWAS database available, including spondylosis, early lumbar disc prolapse (operated), intervertebral disc degeneration, low back pain, sciatica, cervical disc disorders, infection of the intervertebral disc (pyogenic), ankylosing spondylitis (strict definition), ankylosing spondylitis, spinal enthesopathy, scoliosis, spinal instabilities, spinal osteochondrosis, spinal stenosis, kissing spine, kyphosis, lordosis, osteochondrodysplasia with defects of growth of tubular bones and spine, diseases of the spinal cord, cauda equina syndrome, spinal cord benign neoplasm, spinal meninges benign neoplasm, and pain in the thoracic spine. And we classified these diseases according to the site or nature of occurrence into degenerative spinal disorders; inflammatory, infectious, and immunity spinal disorders; structural spinal disorders; spinal cord and neurologically associated disorders; other spinal disorders. The basic information for the GWAS data on the 23 spinal and related diseases is presented in Supplementary Tables S3, and detailed case information and diagnostic criteria for each disease can be found at https://risteys.finregistry.fi/ (30). For example, the diagnosis of IVDD is based on the International Classification of Diseases, specifically ICD-10 (M51), ICD-9722, and ICD-8725 coding standards.

### 2.3 Instrumental variable selection

In order to identify genetic predictors associated with dietary intake and liking, we implemented a stringent quality control procedure. Single-nucleotide polymorphisms (SNPs) that reached the genome-wide statistical significance threshold (*p* < 5.0 × 10^−8^) for association with each dietary intake and liking category were considered as potential instrumental variables (IVs). To ensure compliance with the assumptions of Mendelian randomization (MR), we conducted a linkage disequilibrium (LD) analysis using data from the 1,000 Genomes Project, focusing on individuals of European ancestry. SNPs that did not meet the criteria (*R*^2^ < 0.001, clumping distance = 10,000 kb) were excluded from further analysis. Additionally, SNPs with a minor allele frequency (MAF) below 0.01 were excluded from the analysis. Moreover, to avoid weak instrumental bias in MR analysis, we calculated the F statistic of each IV. F was calculated using the formula:


$$F=\frac{R^{2}\times \left(N-k-1\right)}{k\times \left(1-R^{2}\right)}$$

For each SNPs, *k* = 1, to evaluate the bias of the weak instrument


$$R^{2}=\frac{2\times \beta^{2}\times EAF\times \left(1-EAF\right)}{2\times \beta^{2}\times EAF\times \left(1-EAF\right)+2\times SE^{2}\times N\times EAF\times \left(1-EAF\right)}$$

*R*^2^ represents the proportion of variation interpreted by selected SNPs, N represents the number of participants, EAF represents the effect allele frequency, and *β* is the estimated effect of the SNP to assess its ability to uniquely predict the outcome. Typically, IVs with low F statistics (<10) were removed (31).

### 2.4 Univariable and multivariable Mendelian randomization analyses

We employed the inverse variance weighted (IVW) method as the primary approach for analyzing the MR data (32, 33). To ensure the robustness of our findings, we also conducted several sensitivity analyses using alternative methodologies, including the commonly used MR-Egger regression and weighted median (WM) methods, the former can estimate the causal effects using the slope coefficient of Egger regression (34), while the latter can prevent up to 50% of invalid instrumental variables (IVs) in mendelian randomization analysis (35). Additionally, we utilized the Bayesian weighted Mendelian randomization (BWMR), which addresses violations of the instrumental variable assumptions due to pleiotropy by Bayesian weighted adjustment. This model also considers the uncertainty arising from weak effects due to polygenicity, further enhancing the robustness of causal inference (36). In analyzing the association between alcohol exposure and spinal diseases, and due to the close to 100 instrumental variables (IVs), we introduced the Contamination mixture method (ConMix) to obtain more robust estimates of causal effects (37). Although some of these methods did not yield statistically significant results, we considered the findings positive if the IVW method produced significant results (*p* < 0.05). To assess the impact on spinal and related diseases, we calculated odds ratios (OR) along with 95% confidence intervals (CIs).

Heterogeneity was evaluated using Cochran’s Q test for the IVW and MR-Egger estimates. To investigate potential pleiotropic bias, we utilized the MR-Egger regression technique. When the *p*-value of the heterogeneity test is less than 0.05, the Inverse Variance Weighting (IVW) method with multiplicative random effects will be employed to ensure the robustness of the results. These rigorous analytical approaches were employed to ensure the reliability and validity of the study’s outcomes. To control for the proportion of false positives in multiple testing, we separately performed a false discovery rate (FDR) correction for nominally significant associations between dietary factors and each selected disease. A P_FDR_ value <0.05 was considered as statistically significant. For significant results after FDR correction, indicate a significant association; if the initial results of IVW are significant but become non-significant after FDR correction, it suggest a potential association.

Finally, to uncover potential vertical pleiotropic pathways that could arise from specific dietary-related diseases, we performed multivariable Mendelian randomization (MR) analyses. The analyses were conducted to estimate the causal effect of dietary-related diseases on spinal diseases, including gout, obesity, hypertension, smoking, diabetes and coronary artery disease, after adjusting for five dietary-related diseases or phenotypes, to assess the potential mediating effects of these factors on the risk of spinal diseases. The selection of instrumental variables and the parameter settings for multivariable MR (MVMR) were consistent with those used in univariable MR.

All statistical analyses were conducted using the “TwoSampleMR,” “MendelianRandomization” and “MVMR” package in the R software environment (version 4.3.0).

## 3 Results

### 3.1 Selection of IVs

To investigate the association between dietary factors and the risk of spinal and related diseases, we conducted a MR analysis involving 203 dietary traits and spinal and related diseases. We ensured the use of robust genetic instruments (*p*-values <5 × 10^−8^) to establish the independence of these traits by excluding palindromic SNPs. The instrumental variables exhibited F-statistics that were all significantly greater than 10, indicating the absence of weak instrument bias. These measures were implemented to ensure the reliability and validity of our findings. Detailed information of SNPs for each trait could be found in Supplementary Tables 4, 8.

### 3.2 Exploring the causal effect of dietary intake on spinal and related diseases

**Figure 2:**
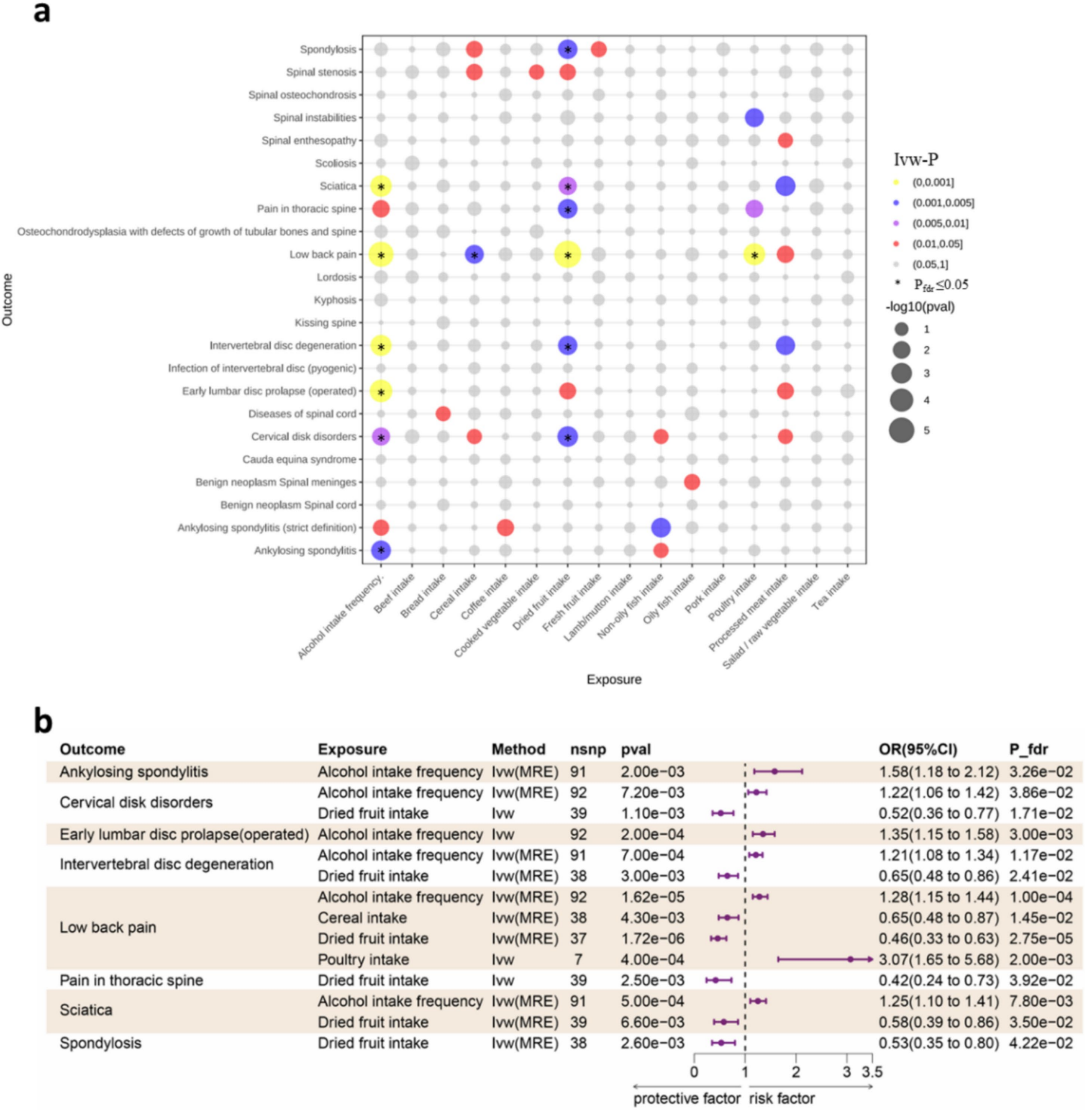
**(a)** Bubble plot for the results of MR analysis. Dot size indicates *p* values generated by IVW method, which be color-coded according to *p* value thresholds of 0.05, 0.01, 0.005, and 0.001. P_fdr_ ≤ 0.05 be marked with an asterisk (*). **(b)** Forest plot of the causality between dietary intake and spinal diseases using IVW method. CI, confidence interval; FDR, false discovery rate; OR, odds ratio; IVW, Inverse variance weighted; MRE, Multiplicative random effects.

When statistically significant results after FDR correction show contradictory trends between dietary intake and liking in the direction of odds ratio (OR), we consider the results to be unreliable and discard them. As is depicted in Figure 2, after FDR adjustment, four dietary intake traits were identified as statistical significance (P_FDR_ < 0.05) and with IVW method used as main method. Alcohol intake frequency increase the risk of ankylosing spondylitis (OR: 1.581, 95%CI:1.182–2.116, P_fdr_ = 0.033), cervical disk disorders (OR:1.224, 95%CI:1.056–1.418, P_fdr_ = 0.037), early lumbar disc prolapse (operated) (OR:1.349, 95%CI:1.153–1.579, P_fdr_ = 0.003), IVDD (OR:1.206, 95%CI:1.082–1.344, P_fdr_ = 0.012) and low back pain (OR:1.283, 95%CI:1.145–1.436, P_fdr_ = 0.0001); poultry intake increase the risk of low back pain (OR:3.067, 95%CI:1.655–5.684, P_fdr_ = 0.002). For the protective dietary factors, dried fruit intake decrease the risk of cervical disk disorders (OR:0.523, 95%CI:0.355–0.771, P_fdr_ = 0.017), IVDD (OR:0.645, 95%CI:0.483–0.862, P_fdr_ = 0.024), low back pain (OR:0.457, 95%CI:0.332–0.630, P_fdr_ = 2.75E-05), pain in thoracic spine (OR:0.416, 95%CI:0.235–0.733, P_fdr_ = 0.039), sciatica (OR:0.582, 95%CI:0.394–0.860, P_fdr_ = 0.035) and spondylosis (OR:0.531, 95%CI:0.352–0.802, P_fdr_ = 0.042); cereal intake decrease the risk of low back pain (OR:0.645, 95%CI:0.478–0.872, P_fdr_ = 0.015). For the 14 major associations identified above, although the statistical significance of the *p*-value may not be evident when using methods other than IVW, our primary focus is on the direction of the odds ratio (OR). When multiple methods consistently demonstrate the effect of dietary intake on spinal diseases, we consider the results reliable. Additionally, we cannot overlook the associations that are no longer significant after FDR correction or those that were not significant before correction. The magnitude of the OR and the width of the confidence interval (CI) used to explain these potential associations also hold significant importance in statistics (38, 39). For sensitive analysis, The intercept of MR-Egger indicated that there was no horizontal pleiotropy which showed the robustness of the results. Detailed information for all MR results and sensitive analysis between dietary intake and spinal diseases could be found in Supplementary Tables 5, 6, 7, respectively.

### 3.3 Exploring the causal effect of food liking on spinal and related diseases

**Figure 3:**
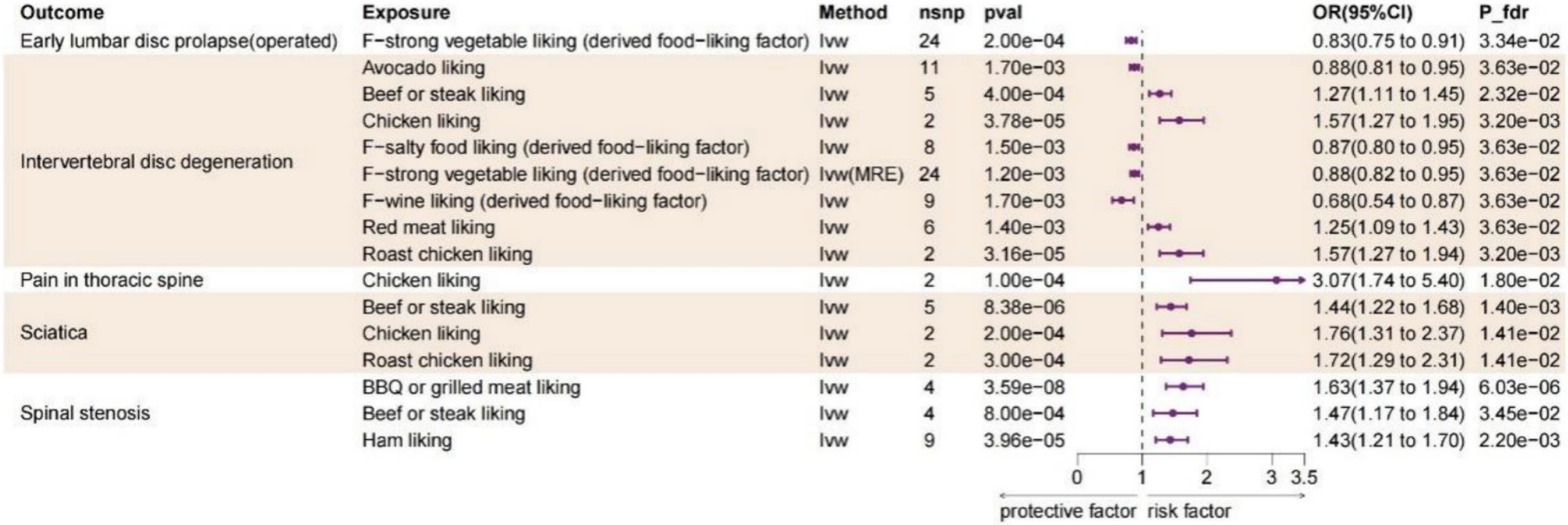
Forest plot of the causality between dietary likings and spinal diseases using IVW method. CI, confidence interval; FDR, false discovery rate; OR, odds ratio; IVW, Inverse variance weighted; MRE, Multiplicative random effects.

Diet liking does not solely reflect daily dietary preferences, but rather encompasses specific hedonic response toward each food, which exhibit a stronger genetic correlation than dietary intake (22). As is depicted in Figure 3, after FDR adjustment, 10 dietary liking traits were identified as statistic significance (P_FDR_ < 0.05) and with IVW method used as main method. BBQ or grilled meat liking increase the risk of spinal stenosis (OR:1.631, 95%CI:1.371–1.942, P_fdr_ = 6.03E-06); beef or steak liking increase the risk of IVDD (OR:1.270, 95%CI:1.112–1.451, P_fdr_ = 0.023), sciatica (OR:1.437, 95%CI:1.225–1.685, P_fdr_ = 0.001) and spinal stenosis (OR:1.470, 95%CI:1.173–1.842, P_fdr_ = 0.035); chicken liking increase the risk of IVDD (OR:1.575, 95%CI:1.269–1.954, P_fdr_ = 0.003), pain in thoracic spine (OR:3.066, 95%CI:1.739–5.403, P_fdr_ = 0.018) and sciatica (OR:1.765, 95%CI:1.313–2.373, P_fdr_ = 0.014); ham liking increase the risk of spinal stenosis (OR:1.431, 95%CI:1.206–1.697, P_fdr_ = 0.002); red meat liking increase the risk of IVDD (OR:1.248, 95%CI:1.090–1.431, P_fdr_ = 0.036); roast chicken liking increase the risk of IVDD (OR:1.572, 95%CI:1.288–2.308, P_fdr_ = 0.014) and sciatica (OR:1.724, 95%CI:1.288–2.308, P_fdr_ = 0.014). For the protective dietary factors, avocado liking decrease the risk of IVDD (OR:0.876, 95%CI: 0.807–0.951, P_fdr_ = 0.036); F-salty food liking (derived food-liking factor) decrease the risk of IVDD (OR:0.873, 95%CI: 0.803–0.949, P_fdr_ = 0.036); F-strong vegetable liking (derived food-liking factor) decrease the risk of early lumbar disc prolapse (operated) (OR:0.825, 95%CI:0.746–0.913, P_fdr_ = 0.033) and IVDD (OR:0.825, 95%CI:0.746–0.913, P_fdr_ = 0.033); F-wine liking (derived food-liking factor) decrease the risk of IVDD (OR:0.682, 95%CI:0.537–0.867, P_fdr_ = 0.036). The identification of the significance of data and sensitivity analysis operations are as previously described. Detailed information for all MR results and sensitive analysis between dietary liking and spinal diseases could be found in Supplementary Tables 9, 10, 11, respectively.

### 3.4 Multivariable MR analysis

Considering the overall significant associations (P_fdr_ < 0.05) and the number of SNPs used as instrumental variables for each exposure, we identified alcohol intake as the most critical risk factor.

We included five diseases or phenotypes associated with alcohol intake, including gout, obesity, hypertension, smoking, diabetes and coronary artery disease, for the MVMR analysis to evaluate the independent effects of alcohol intake frequency on six causality-enriched outcomes (ankylosing spondylitis, cervical disk disorders, early lumbar disc prolapse (operated), IVDD, low back pain and sciatica). The results indicated that after adjusting diabetes (adjusted OR = 1.16 (0.99–1.36); *p* = 0.07), hypertension (adjusted OR = 1.10 (0.93–1.30); *p* = 0.28), and gout (adjusted OR = 1.09 (0.89–1.34)), the causal relationships between alcohol intake and cervical disk disorders was no longer significant. This suggests that these diseases may have potential mediating or direct effects in the progression of cervical disk disorders. All multivariable Mendelian randomization results are presented in Supplementary Table 12. Additionally, beyond focusing on changes in *p*-values, we also consider changes in the OR values in the causal relationship after adjusting diet-related factors; significant attenuation of OR values can also be considered indicative of potential mediating pathways (40, 41). Thus the variation in the OR values further illustrates that hypertension, diabetes, and gout may act as mediating factors in the progression of cervical disk disorders facilitated by alcohol consumption (original OR = 1.22).

## 4 Discussion

The results of this study encompass findings that are both statistically significant and suggestive. In this study, we have collected the most comprehensive GWAS data available, encompassing both objectively measured dietary intake and subjectively dietary likings, as well as a wide range of potential spinal disease outcomes. Our MR analysis has found that degenerative spinal diseases are the most significant outcomes influenced by dietary factors. In terms of dietary intake, the consumption of alcohol and poultry has been identified as significant risk factors, whereas dried fruit and cereal have acted as protective factors. Regarding dietary likings, likings for chicken, roast chicken, beef or steak, red meat, grilled or BBQ meat, and ham was found to be associated with an increased risk of disease. Conversely, likings for vegetable, salty food, avocado, and red wine were found to be protective factors. This comprehensive evaluation highlights the complex interactions between diet and spinal health, emphasizing the importance of dietary choices in managing and potentially preventing spinal diseases. Additionally, to our knowledge, there has not yet been a Mendelian randomization analysis investigating the causal relationships between dietary likings and the risk of spinal diseases. This makes our study the most comprehensive MR analysis to date exploring the causal relationships between dietary factors and spinal health.

This MR analysis has found that Intervertebral Disc Degeneration (IVDD) is the primary spinal disease influenced by dietary factors. IVDD is a common degenerative condition associated with a range of spinal degenerative diseases, including low back pain, sciatica, disc herniation, and spinal stenosis. These diseases pose significant challenges to both patients and society (42). The intervertebral disc (IVD) is composed of the nucleus pulposus (NP), annulus fibrosus (AF), and cartilaginous endplates (CEP), all of which are vital for spinal movement and load distribution. The NP is particularly crucial as it contains a high concentration of water and extracellular matrix (ECM), which are pivotal in sustaining the disc’s functionality. Although the exact mechanisms underlying IVDD are still unclear, numerous studies have highlighted the role of inflammation in the disease’s progression (43–46).

This MR study confirmed that alcohol intake is a risk factor for various degenerative spinal diseases. Some retrospective studies have assessed the relationship between alcohol consumption and lower back pain, but they did not reach definitive conclusions (47, 48). However, a MR study later identified an increased risk of low back pain associated with alcohol intake (42). As IVDD is the most significant cause of lower back pain, the relationship between alcohol consumption and IVDD is receiving increasing clinical attention. A previous study through surveys found a higher prevalence of alcohol abuse in individuals with IVDD compared to the general population; however, the small sample size limited the generalizability of these findings (49). Khatun et al. found that alcohol consumption from adolescence to early adulthood has a detrimental effect on IVDD (50), while Ning Zhang and colleagues reviewed clinical evidence on alcohol consumption and IVDD, finding that moderate drinking could potentially reduce the risk of IVDD (51). However, compared to abstainers, individuals with low to moderate alcohol intake have healthier lifestyles overall, suggesting that the observed effects on IVDD may be confounded by lifestyle factors. High frequency of alcohol consumption could exacerbate inflammatory processes by promoting the expression of inflammatory markers such as TNF-α, leading to a progressively amplified inflammatory cascade and sustained inflammatory state, ultimately causing IVDD. Another possible explanation is that chronic alcohol consumption leads to methylation changes in the DNA of inflammation-related genes (including HERC5), playing a fundamental role in the transcription of inflammatory genes and exacerbating the inflammatory response, ultimately contributing to IVDD (52, 53). Furthermore, excessive alcohol consumption may disrupt hormonal balance, particularly estrogen levels (54). Estrogen has been shown to inhibit IVDD through various mechanisms, including the downregulation of MMP-3 and MMP-13, upregulation of type II collagen, inhibition of the NF-κB signaling pathway, reduction of inflammatory factors IL-1β and TNF-α, suppression of matrix metalloproteinases to reduce catabolism, upregulation of integrins α2 and β1, enhancement of IVD synthetic metabolism, activation of the PI3K/Akt pathway, mitigation of oxidative damage, and promotion of autophagy (55–57). Furthermore, Chronic alcohol consumption not only impairs liver and kidney function but also induces long-term alterations in the gut microbiota, including a significant reduction in Lactobacillus and Bifidobacterium populations and an increase in inflammation-related opportunistic pathogens such as Enterobacteriaceae. Additionally, excessive alcohol intake is closely associated with reduced vitamin D levels and influences the genetic polymorphism of the vitamin D receptor (VDR), which is considered a major contributing factor to low bone mineral density and bone diseases in patients. Vitamin D regulates the immune system through the VDR, enhancing intestinal epithelial barrier integrity, while the gut microbiota modulates immune cell activity by metabolizing short-chain fatty acids (SCFAs). Together, these mechanisms synergistically improve epithelial defense and immune regulation. Deficiency in vitamin D and gut microbiota dysbiosis collectively result in immune dysregulation, promoting the progression of chronic inflammation, which may represent a critical mechanism underlying chronic spinal disease (58–60). In summary, while the mechanisms by which high alcohol intake frequency triggers IVDD are not yet fully understood, our findings can help clinicians educate IVDD patients or those at high risk to reduce their drinking frequency, thus potentially decreasing the incidence of IVDD from a dietary habit perspective.

Likings for food is an individual’s hedonic response to food, a complex trait influenced by genetics, biology, psychology, and environment (61, 62). A study on children aged 7–10 years confirmed that food likings are significantly associated with children’s food choices and intake (63). Hence, discovering or fostering a liking for certain types of food can facilitate targeted dietary interventions. Our MR study identified the liking for wine as a protective factor of IVDD. This could be due to the broad acceptance of wine and its unique components. A cohort study based on the Swedish population showed that, compared to spirits and beer, the group that prefers drinking red wine had a lower incidence of heavy drinking and alcohol abuse (64). Another study on the types of alcoholic beverages consumed by American subpopulations revealed that individuals with a four-year college degree/higher education and higher incomes prefer wine over those with lower educational levels and middle to low incomes (65). This suggests that the group liking wine might be more capable of controlling drinking patterns, having lower drinking frequency and alcohol intake, thereby reducing the risk of developing IVDD. As for the unique components in the wine, for instance, Resveratrol (RSV) is a polyphenolic phytoalexin found in various plants and wine and research indicates that RSV inhibits inflammation and oxidative stress, suppresses apoptosis and aging in NP cells, promotes autophagy, and enhances ECM synthetic metabolism and anti-catabolic metabolism, thereby preventing further degeneration of intervertebral disc cells (66, 67). Although our research highlights the positive role of the liking for wine in reducing the risk of IVDD, excessive drinking has been proven harmful, and merely a liking for wine as a protective factor necessitates further investigation into the specific intake levels of red wine in mitigating IVDD (68).

This MR analysis first revealed that that a liking for chicken, roast chicken, beef or steak, red meat, BBQ or grilled meat and ham as well the intake of poultry has been associated with a higher risk of spinal degenerative diseases. However, to our knowledge, there is currently no research exploring the causal relationship between meat consumption and IVDD. A possible explanation is that the processing of these foods, especially during baking, frying, and grilling, leads to the formation of significant amounts of Advanced Glycation End-products (AGEs), which are ingested into the body with the diet. The accumulation of AGEs can cause tissue damage and deformation and has been shown to be associated with diseases such as diabetes, cardiovascular diseases, kidney diseases, and neurodegenerative diseases (69–71). Studies indicate that as age increases, the deposition of AGEs in the IVD increases, and can be accelerated by diabetes and a high-AGEs diet, leading to the destruction of the annulus fibrosus (AF), nucleus pulposus (NP), and cartilaginous endplate (CEP), ultimately resulting in disc degeneration (72). Divya et al. validated the hypothesis that long-term consumption of a high-AGEs diet leads to gender-specific structural and functional changes in IVD using the mouse model (73). Dietary adjustments and interventions to reduce AGEs could be an effective measure to control the progression of spinal degenerative diseases. The pathogenesis of IVDD is closely associated with elevated levels of pro-inflammatory mediators in the body. A possible reason why the intake of cereal reduces the risk of IVDD is that these foods lower the body’s inflammatory state (74, 75). Furthermore, these foods may improve symptoms associated with musculoskeletal disorders by modulating the immune system and pain perception (76, 77). A liking for vegetables and increased vegetable intake are related and may reduce the occurrence of spinal degenerative diseases by decreasing systemic and central inflammation (78). Research found that supplementing with Omega-3 fatty acids can reduce the serum AA/EPA ratio, alleviate systemic inflammation, and potentially protect against the progression of disc degeneration (79). This aligns with our findings that vegetables favored in the diet, such as spinach, avocados, and asparagus, which are rich in Omega-3 fatty acids, may lower the risk of spinal degenerative diseases.

Our MVMR study results indicate that after including gout, diabetes, and hypertension, the causal relationship between alcohol intake frequency and cervical disc disorders becomes non-significant. This suggests the potential for a mediating effect of alcohol in the development of cervical disc disorders. The primary cause of cervical disc diseases is degenerative changes leading to cervical disc herniation or cervical spondylosis, with other causes including trauma-related acute cervical injuries (80). Alcohol has been considered a risk factor for gout for a long time. A follow-up study of health professionals reported a significant correlation between alcohol intake and increased risk of gout (81). Research by Saki Teramura et al. also identified hypertension and alcohol consumption as risk factors for hyperuricemia or gout in men (82). Furthermore, hypertension and diabetes have been linked to excessive drinking. However, few studies have assessed the correlation between gout, diabetes, hypertension, and cervical disc disorders. Hyperuricemia is a major risk factor for symptomatic gout and can lead to various complications, including gout, metabolic syndrome, coronary artery disease, and type 2 diabetes. Metabolic syndrome is characterized by a cluster of physiological and anthropometric abnormalities, including elevated blood sugar levels, obesity, hypertension, elevated triglycerides, and low HDL cholesterol (83–85). Thus, excessive alcohol intake can lead to complications such as hyperuricemia, gout, hypertension, or diabetes, which may indirectly affect the development of cervical disc disorders. To date, no studies have explored the relationship between hyperuricemia and cervical disc disorders, and this hypothesis requires further investigation to be confirmed. Furthermore, our MVMR analysis included only a subset of diseases. A systematic exploration is required to understand the interrelationships between more diet-related diseases and spinal disorders.

Furthermore, our MR study did not find statistically significant causal relationships between dietary factors and other spinal diseases unmentioned. Due to space constraints, we were unable to discuss all the spinal diseases included in our study; however, we can provide an illustrative example. Previous research by Benjamin et al. demonstrated that obesity, particularly when accompanied by diabetes, is associated with a higher incidence of *Staphylococcus aureus* infections and poses a risk factor for inflammatory spinal discitis (86). Consequently, we consider that metabolic diseases such as obesity and diabetes may have a more significant role in discogenic infectious diseases compared to merely dietary factors. Sarcopenia and degenerative changes in the back muscles have been identified as risk factors for adult degenerative spinal deformities with sagittal imbalance (87). A retrospective cohort study suggested that infections might lead to bone destruction, thus leading to spinal deformities (88). However, no studies have established a correlation between diet and spinal deformity. Moreover, our MR findings indicate that there is no causal relationship between dietary factors and spinal deformities. It is likely that dietary factors do not significantly influence the development of spinal deformities, which are more closely related to genetic susceptibility, daily posture, and spinal activity state.

## 5 Restrictions

Since our Mendelian randomization study was based on existing GWAS data available, the reliability of our conclusions is largely dependent on the quality of the GWAS data used. Additionally, due to limitations in the sources of the GWAS data, our study was confined to European populations. In the future, we hope to include more diverse populations and more comprehensive GWAS data covering a wider range of exposures and outcomes to derive more extensive and credible causal conclusions. Considering the limitations of MR, its findings should be applied and generalized with great caution and require further external validation. Nevertheless, our study provides preliminary causal evidence linking dietary factors to spinal diseases.

## 6 Conclusion

In summary, our study has extensively delved into the impact of 16 dietary intake and 187 dietary likings on 23 spinal diseases, and incorporated six diseases highly correlated with diet to differentiate confounding factors and explore mediating effects. Our findings reveal that degenerative spinal diseases are most significantly influenced by dietary factors, with alcohol intake emerging as the most significant risk factor. Additionally, the development of cervical disc disorders may be associated with certain diet-related diseases. These insights, providing direct or indirect causal evidence, enhance our understanding of how dietary factors can promote or protect against the occurrence and progression of spinal diseases. This knowledge not only enables targeted interventions to prevent or alleviate the progression of spinal diseases based on population dietary intake but also holds promise for predicting future risk of spinal diseases based on an individual’s current dietary intake and likings, allowing for timely interventions.

## Data Availability

The original contributions presented in the study are included in the article/Supplementary material, further inquiries can be directed to the corresponding authors.

## Footnotes

^1^ https://gwas.mrcieu.ac.uk/

^2^ https://biobank.ctsu.ox.ac.uk/crystal/label.cgi?id=100050

^3^https://r10.finngen.fi/, Public release: 18 Dec 2023.
